# Beyond vascular recanalization: precision medicine in cervical artery dissection guided by multiple mechanisms and multimodal imaging for brain functional recovery

**DOI:** 10.3389/fphys.2025.1695764

**Published:** 2026-01-30

**Authors:** Tao Li, Linna Li, Kexin Zhao, Jiaqi Dai, Xueliang Tian, Xuanxiao Zhang, Shuo Yin, Wenjing Lan, Hongwei Zhou

**Affiliations:** 1 Department of Radiology, The First Hospital of Jilin University, Changchun, Jilin, China; 2 Department of Endocrinology, The First Hospital of Jilin University, Changchun, Jilin, China; 3 Department of Breast Surgery, General Surgery Center, The First Hospital of Jilin University, Changchun, Jilin, China

**Keywords:** cervical artery dissection, endovascular therapy, hemodynamics, high-resolution MRI, ischemic stroke, pathomechanisms, precision medicine, recanalization

## Abstract

Cervical artery dissection (CAD), a critical etiological contributor of stroke in young adults, exerts direct influence on neurological prognosis through its vascular recanalization outcomes. However, pathological heterogeneity and ongoing controversies surrounding treatment strategies hinder the optimization of clinical decision-making; its prognosis is often not favorable. At present, there are a relative paucity of studies on recanalization of CAD. This review provides a concise overview of the pathological mechanisms and clinical challenges associated with CAD, along with recent advancements in advanced imaging modalities and clinico-anatomical classification systems. Furthermore, we critically evaluate current therapeutic paradigms and factors influencing recanalization while elucidating potential biological mechanisms underlying vascular restoration. A systematic analysis of translational utility in animal models is presented. Finally, based on the latest research progress in CAD and vessel recanalization, prospects are outlined aiming to establish a theoretical foundation for developing personalized and precise therapeutic approaches targeting CAD recanalization from multidimensional perspectives, and to offer reference for subsequent research.

## 1 Introduction

Cervical artery dissection (CAD), a prototypical pathological alteration of vascular wall structural integrity, involves intimal tearing or vasa vasorum rupture-induced intramural hematoma formation in carotid arteries (including internal carotid and vertebral arteries). This pathological process may secondarily lead to luminal thrombosis, vascular stenosis/occlusion, or pseudoaneurysm formation, representing a significant risk factor for ischemic stroke in young and middle-aged populations. Recent epidemiological data demonstrate that the incidence of spontaneous CAD increased nearly fourfold between 2002 and 2020. Notably, incidence rates in females rose more than twelvefold, revealing marked sex disparities. The current incidence of spontaneous CAD reaches 8.93 per 100,000 person-years, nearly triple previous estimates. Whilst CAD accounts for merely 2% of all ischemic strokes, its proportion escalates to 15%–25% among patients under 50 years old, exhibiting high mortality, disability, and recurrence rates—a triad of severe clinical characteristics that pose substantial threats to public health (Béjot et al., 2014; Debette and Leys, 2009; Griffin et al., 2024; Lee et al., 2006; Putaala et al., 2009; Schipani et al., 2024; Yaghi et al., 2024b).

The etiological spectrum of CAD exhibits significant heterogeneity, with approximately 90% of traumatic dissections originating from minor mechanical injuries during routine activities like cervical massage, weight training, or violent coughing (Keser et al., 2022). Multivariate analyses implicate multiple contributing factors including host genetic predisposition (such as Ehlers-Danlos syndrome type IV), vascular structural abnormalities (elongated styloid processes, vascular tortuosity), hormonal fluctuations (pregnancy, oral contraceptive use), and environmental influences (recent infections, smoking history, and educational level) (Debette and Leys, 2009; Kellert et al., 2018; Kim et al., 2016; Muthusami et al., 2013; Ogura et al., 2015; Rist et al., 2011; Subedi et al., 2017). Genetic susceptibility, in particular, appears to play a potentially influential role in CAD pathogenesis. Robust evidence supports associations between CAD and specific genetic disorders, especially monogenic connective tissue diseases including vascular Ehlers-Danlos syndrome, Marfan syndrome, osteogenesis imperfecta, and Loeys-Dietz syndrome (Debette and Markus, 2009; Germain, 2002). Molecular genetic studies further reveal that methylenetetrahydrofolate reductase (MTHFR) C677T polymorphism and PHACTR1 rs9349379 [G] allelic variants significantly elevate CAD risk (Debette et al., 2015; Luo et al., 2014), though the correlation between these risk factors and vascular recanalization outcomes requires deeper investigation. Current research gaps persist regarding CAD-specific triggering factors and unique pathophysiological mechanisms.

The current evidence base suffers from scarcity of high-quality evidence-based studies, with most literature comprising case reports and small retrospective series lacking multicenter randomized controlled trial validation (Keser et al., 2022). Whilst technological advances in high-resolution MRI and CT angiography have markedly improved CAD detection rates, this has created growing tension with demands for precision medicine (Hakimi and Sivakumar, 2019). Contemporary treatment strategies (including thrombolysis, antiplatelet/anticoagulant therapy, and endovascular interventions) demonstrate substantial therapeutic heterogeneity in clinical practice, with 30%–40% of patients failing to achieve expected outcomes, highlighting critical knowledge gaps in recanalization mechanisms and influencing factors. As the core biological marker for treatment response evaluation, vascular recanalization involves multiple pathophysiological processes including thrombus dissolution, intimal repair, and hemodynamic remodeling. Yet systematic investigations into recanalization time windows, influencing factors (dissection type, thrombus burden, collateral status), and intervention strategies remain notably deficient.

This review synthesizes translational evidence across pathological mechanisms, imaging evaluation systems, and treatment strategies to propose a hemodynamic and molecular classification-guided therapeutic framework. Our objectives are twofold: providing theoretical foundations for optimizing personalized treatment decisions while delineating future directions for translational research.

## 2 Pathomechanisms and clinical challenges

### 2.1 Pathological mechanisms

The pathogenesis of cervical artery dissection (CAD) retains multiple unresolved aspects. Current academic consensus identifies mechanical injury to vascular wall structure as the primary etiology (Debette et al., 2011). When cervical arteries undergo abnormal tensile, rotational, or compressive forces, intimal layer continuity may be disrupted, creating mobile intimal flaps. Subsequent blood entry through damaged intima into the vessel wall interstitium progressively forms characteristic dissecting pseudo-lumens. Notably, some cases manifest as intramural hematomas secondary to vasa vasorum rupture within the internal elastic lamina without typical intimal tear features (Bond et al., 2021).

Imaging studies reveal these lesions induce significant vascular morphological changes: approximately 25% of internal cervical artery dissection (ICAD) patients develop complete luminal occlusion, while vertebral artery dissection (VAD) shows even higher complete occlusion rates reaching 50% (Salehi Omran, 2023). Concurrently, 56% of ICAD and 39% of VAD cases exhibit varying degrees of non-occlusive stenosis. When vascular injury extends to the adventitial layer, potentially hazardous pseudoaneurysmal dilatation may occur—a pathological change attributable either to hematoma extension into the adventitia or micro-ruptures of the external elastic lamina. Some scholars hypothesize that spontaneous CAD (sCAD) primarily involves outer arterial structures through outside-in mechanisms. This process initiates with degenerative changes at the medial-adventitial junction, accompanied by neovascularization from vasa vasorum-derived capillaries. Subsequent leakage from these nascent capillaries releases blood cells into connective tissue, forming microhematomas along medial/adventitial junctions that ultimately cause medial-adventitial separation (Völker et al., 2011).

From a pathophysiological perspective, CAD clinical manifestations primarily correlate with three mechanisms: First, mass effects from pseudo-lumen expansion may stimulate perivascular nerve plexuses to produce local symptoms. Second, turbulent flow at dissection sites promotes unstable thrombus formation, with subsequent embolic migration potentially causing distal arterial embolism (Bond et al., 2021; Debette et al., 2011). Third, severe luminal stenosis or occlusion may induce cerebral hypoperfusion, where compensatory collateral circulation—for example, Willis circle—becomes critical for maintaining cerebral blood flow (Wang et al., 2020). Particularly noteworthy, thromboembolic complications represent the most dangerous pathological outcome due to high disability rates, with occurrence closely related to hemodynamic disturbances at dissection sites. CAD is now recognized as a multifactorial disease resulting from synergistic interactions between genetic abnormalities and environmental factors. Research is in desperate need to enhance understanding of environmental and genetic predispositions for CAD while evaluating long-term outcomes.

In addition, carotid atherosclerosis is a common cause of carotid artery stenosis. Arterial dissection is essentially a separation between the layers of the vascular wall. On imaging, it often presents as crescent-shaped or ring-shaped intramural hematomas, intimal flaps, double-chambered signs, and flame-like occlusions (Rodallec et al., 2008). The lesion usually starts beyond the carotid bulb and extends into the brain. Atherosclerosis is caused by the formation of lipid plaques beneath the intima and leads to stenosis. On imaging, it mainly manifests as localized eccentric or centripetal plaques, and the plaques may show enhancement after enhancement, with the stenotic areas mostly located at the carotid bifurcation and the starting segment (Cai et al., 2025). Although carotid dissection and atherosclerosis are two independent diseases, they have an important association in clinical practice.

### 2.2 Clinical challenges

Clinical management of CAD recanalization faces significant challenges due to the conflict between pathological complexity and personalized treatment demands. Spontaneous recanalization rates vary widely (30%–60%) without reliable predictive biomarkers, hindering standardization (Babaoglu et al., 2014). While biomarkers show promise for thromboembolic risk stratification in acute phases—potentially optimizing antithrombotic strategies—clinical use is limited by insufficient validation (Babaoglu et al., 2014; Pabinger and Ay, 2009).

Diagnostic delays are problematic, with ∼3.3% of CAD patients misdiagnosed within 2 weeks pre-symptom onset, potentially missing critical early intervention opportunities (Liberman et al., 2020). Therapeutic selection is complicated by antithrombotic therapy’s “double-edged sword” effect: reducing stroke risk while potentially inhibiting endothelialization and increasing dissection extension risk (Peng et al., 2017). Recent RCTs show comparable short-term outcomes for anticoagulants vs antiplatelets, but small sample sizes limit reliability.

Endovascular advances offer opportunities. A 2023 multicenter study confirmed stenting achieves 100% immediate patency in conservatively unresponsive patients, with 1-year stroke recurrence rates similar to surgery (Feng et al., 2024). However, such intervention measures carry the risk of stent thrombosis, and may require anticoagulation therapy for the patients, thereby increasing the risk of bleeding.

Imaging limitations compound decision-making. CT/MRI exhibit 20%–30% false-negative rates for dynamic intramural hematoma evolution. Computational fluid dynamics models can predict 90% of shear stress abnormality regions but require large-scale validation before clinical use (Hakimi and Sivakumar, 2019).

Long-term outcome management suffers evidence gaps. A retrospective analysis of 1017 CAS procedures (907 patients) showed 14% 2-year and 17% overall in-stent restenosis rates (Lai et al., 2023). Crucially, even after successful mechanical thrombectomy, microcirculatory dysfunction (reduced capillary density, insufficient collateral flow) persists in ∼30% of cases, contributing to neurological deficits (Freitas-Andrade et al., 2020; Sperring et al., 2023).

These unresolved issues constrain therapeutic optimization. Future research must integrate multimodal imaging with exploration of molecular pathogenesis and recanalization mechanisms for precision medicine breakthroughs.

## 3 Imaging evaluation and classification

### 3.1 Advances in multimodal imaging

Current imaging for carotid artery dissection (CAD) recanalization includes MRI, CT, DSA, and ultrasonography. Traditional imaging modalities have established roles but also possess inherent limitations.

Digital subtraction angiography (DSA) remains the gold standard for CAD diagnosis, providing high-resolution visualization of lumen morphology, intimal flaps, and hemodynamics. However, its invasive nature, radiation exposure, and contrast-related risks limit its use as a first-line screening or follow-up tool, and it is now primarily reserved for planned interventions (Hakimi and Sivakumar, 2019). Computed tomography angiography (CTA) allows for rapid assessment with high sensitivity and specificity for dissection-related signs such as pseudoaneurysms, intimal flaps, and stenosis. Nevertheless, the slice thickness of conventional CTA (approximately 0.6 mm) poses limitations in depicting subtle dissection components (Keser et al., 2024; Rajendran et al., 2022). Magnetic resonance angiography (MRA) and conventional MRI sequences (e.g., T1-weighted imaging) offer non-invasive visualization of the vessel wall and lumen. However, their ability to detect acute intramural hematoma can be constrained by signal-to-noise ratio and motion artifacts, and they offer limited quantitative hemodynamic assessment (Hakimi and Sivakumar, 2019). Carotid ultrasonography enables dynamic, non-invasive monitoring and is widely used for follow-up, but it has relatively low spatial resolution, is operator-dependent, and provides poor visualization of the skull base segments (Baracchini et al., 2010; Strunk et al., 2021).To overcome these limitations, several novel and more experimental imaging techniques are being explored and applied.

High-resolution MRI (3D HRMRI) provides submillimeter isotropic resolution, clearly visualizing intramural hematomas, intimal flaps, and luminal stenosis while dynamically monitoring hematoma absorption and luminal remodeling—critical for guiding therapy timing. Its high spatial resolution enables precise identification of irregular intimal surfaces and mural thrombi, thereby improving ischemic stroke risk stratification (Hunter et al., 2012; Luo et al., 2016; Shao and Wang, 2025; Zhang et al., 2015; Zhu et al., 2022). Four-dimensional flow MRI (4D Flow MRI) permits quantitative hemodynamic analysis (e.g., wall shear stress, pressure gradients). When combined with 3D visualization, it facilitates post-recanalization stroke risk assessment (Kawano et al., 2022; Peper et al., 2020). Three-dimensional T1-SPACE sequences help elucidate the relationship between occlusion morphology and recanalization success (Chao et al., 2021).

Diffusion-weighted imaging (DWI) demonstrates exceptional sensitivity and specificity for crescent-shaped hyperintense signals in carotid hematomas without false positives. It provides immediate evidence of dissection, facilitating rapid initiation of antithrombotic therapy and planning for endovascular intervention (Adam et al., 2020). Notably, DWI reveals that stroke occurrence and patterning remain independent of dissection stenosis severity (Naggara et al., 2012). Susceptibility-weighted imaging (SWI) and motion-sensitized driven equilibrium (MSDE) improve hematoma detection by suppressing blood flow signals (Hakimi and Sivakumar, 2019).

In CT imaging, photon-counting CTA achieves ultrathin slices of 0.2 mm, significantly enhancing the visualization of dissection components compared to conventional CTA (0.6 mm) (Keser et al., 2024; Rajendran et al., 2022). In the ultrasound domain, intravascular ultrasound (IVUS) greatly enhances the precision of interventional procedures through real-time assessment of the lumen and vessel wall (Zlatancheva et al., 2022). Furthermore, contrast-enhanced ultrasound can provide evidence to guide anticoagulation decisions during thrombus remodeling, which may persist for up to 2 years (Baracchini et al., 2010; Strunk et al., 2021).

These advances pave the way for an integrated anatomical-functional-molecular evaluation system. Combining HR vessel wall MRI, photon-counting CTA, 4D flow MRI, and IVUS holds promise for guiding comprehensive CAD recanalization management (See Table 1).

**TABLE 1:** Anatomical-functional-molecular integrated imaging evaluation system.

Modality	Anatomical resolution	Functional assessment	Molecular features	Clinical value
HR-VW-MRI	200 μm	Intramural hematoma staging	Fibrin deposition	Recanalization potential evaluation
Photon-counting CTA	0.4 mm^3^	Pseudo-lumen volume measurement	Calcified plaque identification	Interventional pathway planning
4D flow MR	1.5T	Shear stress distribution	Hemodynamic parameters	Thrombosis risk prediction
IVUS	100–150 μm	Dynamic dissection morphology assessment	Intramural hematoma composition analysis	Recanalization stratification and interventional guidance

Imaging for internal carotid artery (ICA) and external carotid artery (ECA) dissections aims to identify the intimal flap, double lumen, and intramural hematoma, but differs in focus (See Table 2). ICA dissection requires high diagnostic precision due to stroke risk, making high-resolution MRI vessel wall imaging the gold standard for definitive wall characterization. ECA dissection imaging prioritizes anatomical clarity of its tortuous branches, where CTA is often the preferred initial choice for its superior 3D spatial resolution. DSA is reserved for problem-solving or intervention in both, while ultrasound has limited utility, mainly for proximal screening.

**TABLE 2:** Imaging modalities for the diagnosis of CAD: A comparison between ICA and ECA dissections.

Imaging method	Application and value in internal carotid artery dissection	Application and value in external carotid artery dissection
High-resolution MRI/MRA	Gold standard. Best for displaying intramural hematoma and vessel wall structure	Highly effective, particularly suitable for assessing intramural hematoma, but limited in visualizing small branches
CTA	First-line/Emergency preferred. Fast, comprehensive, good for displaying lumen and calcification	First-line/Preferred. Superior to MRA for anatomical display of ECA and its branches
DSA	Traditional gold standard, now primarily used during treatment. Highest resolution, but invasive	Used for difficult diagnoses or interventional treatment. Provides the most precise lumen morphology
Ultrasound	Useful screening and follow-up tool, but with limited sensitivity and specificity	Limited screening tool, only assesses the proximal main trunk

### 3.2 Clinical significance of classification systems

Classification systems for cervical artery dissection hold substantial value for guiding clinical management and prognostic evaluation. Early research established that dissection typing correlates closely with clinical manifestations and healing rates, providing theoretical foundations for optimized treatment strategies (DiMusto et al., 2017).

**FIGURE 1:**
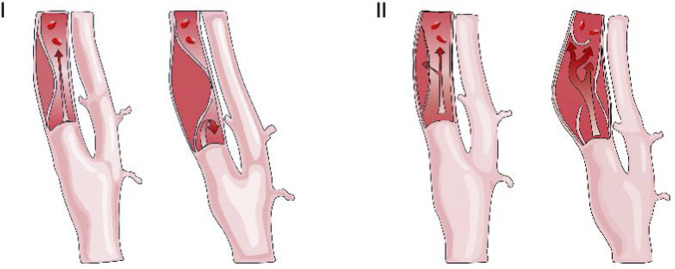
Borgess classification of carotid artery dissection.

The 2013 Borgess classification proposed by Perry and Al-Ali (2013) categorizes spontaneous CAD (sCAD) into type I (absence of intimal tear) and type II (presence of intimal tear) based on imaging characteristics of carotid intimal defects (See Figure 1). Their studies demonstrated significantly higher vascular healing rates in type I dissections receiving antithrombotic therapy compared to type II, suggesting classification-guided medication selection. However, no direct association emerged between classification and ischemic stroke risk—stroke occurrences predominantly clustered within the first post-dissection week, potentially related to sex factors (Reyes et al., 2023).

**FIGURE 2:**
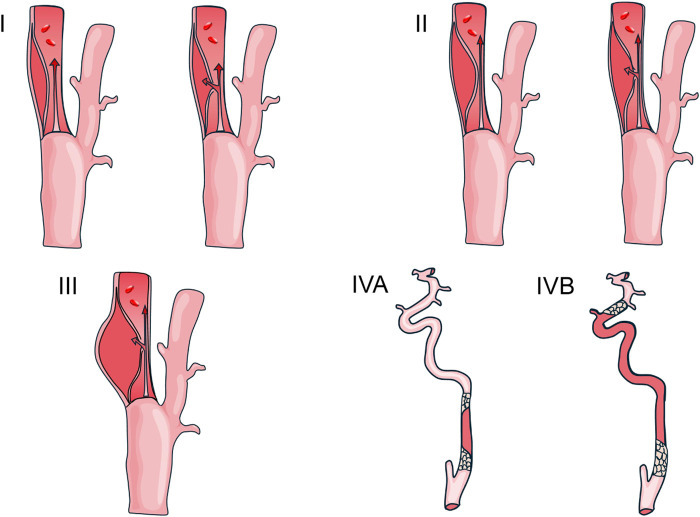
Classification of carotid artery dissection in 2024.

With expanding applications of endovascular therapy (EVT), limitations of traditional classifications for modern decision-making have become apparent. In 2024, a more refined imaging classification system was proposed, categorizing sCAD into four types: type I (intramural hematoma or dissection with <70% luminal stenosis), type II (≥70% stenosis), type III (dissecting aneurysm), type IVA (extracranial carotid occlusion), and type IVB (tandem occlusion) (See Figure 2) (Zhou B. et al., 2024). This system integrates stenosis severity, morphological features, and hemodynamic status to define intervention principles for each subtype: type I—antithrombotic therapy recommended for stroke prevention; types II-IVA—consideration of non-urgent EVT for hemodynamic improvement; type IVB—requiring emergent recanalization due to tandem lesions.

This stratified approach not only enhances therapeutic precision but also reveals EVT’s short-term potential for sCAD management—though long-term efficacy requires validation through large-scale prospective studies. In addition, proposals have emerged for developing recanalization scoring systems based on classification features, potentially adapting models from carotid occlusion recanalization scoring systems to quantify characteristics for optimized decision-making (Jin et al., 2023).

The evolution of CAD classification from purely anatomical descriptions toward integrated treatment-oriented frameworks provides critical structure for personalized therapy and outcome prediction.

## 4 Evidence-based translation of treatment strategies

### 4.1 Intravenous thrombolysis

Intravenous thrombolysis (IVT) for acute ischemic stroke secondary to extracranial/intracranial artery dissection has garnered support from multiple studies (Engelter et al., 2009). Current evidence confirms that intravenous alteplase or tenecteplase administration within 4.5 h of symptom onset significantly improves outcomes in CAD patients, with safety profiles comparable to non-dissection stroke etiologies. Although theoretical concerns exist regarding potential thrombolysis-induced intimal injury increasing risks of subarachnoid hemorrhage or cerebral hematoma in intracranial arterial dissections, large-scale studies demonstrate equivalent bleeding risks between dissection and non-dissection cohorts, establishing IVT as a safe and effective CAD treatment (Bernardo et al., 2019; Engelter et al., 2012; Lin et al., 2016).

For CAD-related acute ischemic stroke (CAD-AIS), while IVT has not conclusively demonstrated superior neurological recovery, its safety, manifested by intracranial hemorrhage rates matching non-CAD patients, has been validated, leading current guidelines to recommend IVT for all eligible patients (Shu et al., 2025).

### 4.2 Selection and time window for antithrombotic therapy

Optimal antithrombotic strategies for CAD-related stroke remain controversial. Current guidelines recommend anticoagulant or antiplatelet agents to reduce thromboembolic risks and prevent stroke recurrence, though relative efficacy between these approaches remains unclear. Two multicenter, open-label, blinded-endpoint studies demonstrated equivalent ischemic event prevention between aspirin and anticoagulants in CAD patients, with overall low recurrent ischemia rates suggesting treatment effects may be independent of antithrombotic class (Georgiadis et al., 2009; Markus et al., 2019).

Despite comparable efficacy and safety between anticoagulant and antiplatelet therapies for preventing ischemic events in CAD, clinical practice often favors anticoagulation due to its theoretical advantages in suppressing thrombus formation at dissection sites—potentially offering superior thromboembolic risk reduction. However, while anticoagulation may provide greater ischemic stroke prevention benefits, this comes with elevated major bleeding risks, necessitating careful individualization weighing ischemic prevention against hemorrhagic complications (Yaghi et al., 2024a). Current evidence indicates no significant overall safety differences between strategies, though optimal selection for specific patient subgroups requires further validation.

Guideline recommendations emphasize individualized antithrombotic strategies based on bleeding risks, dissection morphological characteristics, and dynamic monitoring. Imaging studies confirm typical arterial dissection healing cycles of 3–6 months, offering anatomical rationale for treatment duration. The 2017 European Stroke Organization consensus recommends an initial treatment period of 6–12 months, with discontinuation considered following successful recanalization and stabilization of symptoms—though patients with residual dissecting aneurysms or significant stenosis require prolonged courses (Ahmed et al., 2017).

Earlier American Heart Association guidelines, based on multicenter data analyses, proposed baseline 3–6 months treatment durations (Kleindorfer et al., 2021; Powers et al., 2019). A large clinical study (*n* = 1,390) revealed only 1.4% ipsilateral stroke/TIA incidence during antithrombotic treatment (≤6 months), compared to 3.4% overall event rates during subsequent observation (>6 months)—though no statistical differences emerged between treatment strategies (antiplatelet 3.3%, anticoagulant 2.0%, untreated 4.5%). Notably, only one stroke event during extended follow-up correlated with dissection recurrence (Pezzini et al., 2022).

Updated AHA guidelines stress comprehensive consideration of vascular repair status, hemodynamic features, and comorbidity profiles, similarly recommending minimum 3–6 months baseline therapy. For patients with anatomical residual lesions or high recurrence risks, periodic imaging-guided treatment adjustment is advised to maintain dynamic risk-benefit balance (Yaghi et al., 2024b). In summary, a 3–6 months antithrombotic treatment window appears most clinically appropriate.

### 4.3 Advances in endovascular therapy

Endovascular management of cervical artery dissection (CAD) increasingly emphasizes device innovation to address specific limitations, particularly in complex scenarios such as dissections with pseudoaneurysms. While stent implantation is often required to restore lumen integrity and prevent thromboembolism, the ideal stent design remains elusive, necessitating careful selection based on anatomical and pathological features.

Recent advancements highlight the role of novel stent constructs. For high cervical and long-segment extracranial dissections, braided stents like the Leo Plus stent demonstrate favorable technical success, long-term patency, and efficacy in promoting pseudoaneurysm occlusion (Lu et al., 2024). This aligns with evidence supporting stent placement as safe and effective for symptomatic extracranial internal carotid artery dissection, especially with high-grade stenosis or expanding pseudoaneurysm (Szmygin et al., 2025). In cases involving carotid artery reconstruction or dissecting aneurysms, covered stents and dual-layer micromesh stents (e.g., CASPER) offer solutions by providing immediate flow diversion and vessel wall reconstruction, effectively excluding pseudoaneurysms while maintaining patency (Sun et al., 2025; Matsukawa et al., 2022).

Technical refinements continue to evolve. Proximal embolic protection under flow arrest appears safe and feasible for symptomatic internal carotid dissections, reducing intraprocedural risk (Bruno et al., 2025). However, challenges persist, such as stent thrombosis; early administration of P2Y12 antagonists and adjunctive angioplasty are associated with higher 24-h stent patency, which is itself linked to better functional outcomes (Allard et al., 2023). Furthermore, bailout techniques have been developed to manage rare but serious intraprocedural complications, such as entanglement between stent retrievers and carotid stents, preserving vessel patency and patient safety (Malik et al., 2023).

Current consensus reserves intervention for cases refractory to medical therapy or those at high risk, aiming to rapidly restore flow. Future progress hinges on the development of dedicated dissection stents with enhanced conformability, thromboresistance, and smarter protection systems, supported by randomized trials to optimize patient selection and technical pathways.

## 5 Determinants of vascular recanalization

Mechanistic and influencing factor studies of CAD recanalization reveal multiple interrelated determinants. Research indicates hypertension comorbid with diabetes and obesity may elevate stroke and adverse outcome risks (Tao et al., 2015). Paradoxically, the specific impact of hypertension on recanalization remains a subject of debate—some studies suggest inhibitory effects (Wadhwa et al., 2023), while others report no correlation in younger cohorts (Arnold et al., 2009), possibly reflecting insufficient sample sizes. A meta-analysis comparing 2185 CAD patients with 3,185 healthy controls confirmed significant hypertension associations (Abdelnour, 2022; Abdelnour et al., 2022), supporting plausible hypertension-mediated recanalization impairment.

Moreover, recanalization processes exhibit clear temporal dependence, with approximately 80% occurring within 6 months post-symptom onset—independent of dissection location, morphology, or vascular pattern (Arauz et al., 2010; Patel et al., 2020). Low baseline NIH Stroke Scale scores, non-occlusive lesions (e.g., hypoechoic intramural hematomas), and early pharmacological intervention, such as antiplatelet therapy, correlate with higher recanalization rates and favorable neurological outcomes (Huang et al., 2020; Wadhwa et al., 2023), whereas complete vascular occlusion (particularly involving entire internal carotid artery segments) significantly reduces recanalization success (Hauck et al., 2011).

Chen et al. (2016) developed an angiographic feature-based scoring system (84.7% sensitivity, 67.9% specificity) for predicting occluded vessel recanalization, subsequently validated by independent studies. Key positive predictors include short occlusion duration, tapered ICA stump morphology, and patent distal ICA lumen *via* collateral filling—providing valuable insights for CAD recanalization strategies.

Regarding treatment strategies, some researchers propose anticoagulants may expand intramural hematomas and impede recanalization (Vicenzini et al., 2011), while others observe non-significant trends toward higher complete recanalization rates with anticoagulation (Nedeltchev et al., 2009). The multicenter prospective randomized CADISS trial found equivalent 1-year recanalization rates between anticoagulant and antiplatelet treatments (Markus et al., 2019), and the conclusion drawn by later researchers was consistent with this (Huang et al., 2020). Dual antiplatelet regimens and intravenous heparin efficacy require further validation, while endovascular therapy (EVT) for non-acute occlusions becomes increasingly feasible with technological advances—though distal embolism risks demand vigilance (Ma et al., 2024). Future integration of clinical, imaging, and molecular biomarkers into recanalization scoring systems may optimize intervention timing and modality selection.

## 6 Biological mechanisms of vascular recanalization

### 6.1 Hemodynamic factors

Research suggests CAD development correlates closely with localized wall stress abnormalities, where head movements may induce elevated intramural stress distal to carotid bulbs. Patients with abnormal vascular function/anatomy or chronic excessive stress exposure may experience accelerated pathological progression (Callaghan et al., 2011). Ischemic strokes in these patients frequently originate from arterial embolism or hemodynamic compromise—where anticoagulation significantly reduces embolic risks, while partial recanalization (without complete restoration) suffices to improve perfusion and reduce infarction risks.

Clinically, combined CT perfusion (CTP) and CTA enables dynamic intracranial hemodynamic assessment in acute dissection patients, informing endovascular recanalization decisions (Hakimi and Sivakumar, 2019). Flow-sensitive 4D MRI permits noninvasive analysis of wall shear stress (WSS) distribution at carotid bifurcations, revealing associations with geometric features (diameter ratios, bifurcation angles, tortuosity), stenosis severity, and surgical interventions—facilitating atherosclerosis risk localization and post-treatment WSS redistribution monitoring (Markl et al., 2010).

Computational fluid dynamics modeling demonstrates that stenosis exceeding 70% elevates WSS to 15–25 Pa (normal range: 1.5–3 Pa), increasing endothelial apoptosis 3.2-fold while driving smooth muscle phenotypic switching and vascular remodeling through PI3K/Akt pathways. Concurrently, low WSS and abnormal pressure gradients downstream of stenoses may directly contribute to acute ischemic stroke pathogenesis—offering novel hemodynamic targets for optimizing carotid stenosis management strategies.

### 6.2 Molecular mechanisms

Molecular regulation of CAD recanalization potentially involves multilevel networks encompassing genetic predisposition, extracellular matrix (ECM) homeostasis disruption, epigenetic regulation, and vascular smooth muscle cell (VSMC) phenotypic switching. Genome-wide association studies implicate PHACTR1 and collagen family gene, namely, COL12A1, variants in CAD pathogenesis—possibly mediated through endothelin-1 (EDN1) expression and collagen structure modulation (Debette et al., 2015; Gupta et al., 2017; Traenka et al., 2019).

Proteomic analyses of recurrent CAD further highlight ECM dysfunction as central to disease mechanisms: collagen/elastin fiber structural abnormalities (COL12A1, MFAP5), desmosomal protein (JUP)-mediated endothelial adhesion defects, and cystatin B deficiency-induced ECM hyperdegradation collectively constitute a “ECM homeostasis disruption-endothelial repair impairment” dual pathological axis, compromising vascular wall mechanical stability and re-endothelialization (Debette et al., 2014; Garrod et al., 2005; Mayer-Suess et al., 2020; Traenka and Debette, 2020; Ulbricht et al., 2004).

Simultaneously, microRNAs (e.g., miR-144-3p, miR-124) may drive VSMC transition from contractile to synthetic phenotypes *via* targeted regulation of elastin synthesis (TE) or SP1 signaling—activating MAPK pathways (P38/JNK) to exacerbate vascular structural damage, potentially representing molecular pathways for CAD recanalization (Cao et al., 2022; Ding et al., 2022; Qi et al., 2018; Small and Olson, 2011; Tang et al., 2017; Yang et al., 2022). Additionally, β-blockers may improve blood pressure control and vascular remodeling through β-adrenergic receptor/transforming growth factor-β1 axis modulation, providing genetic rationale for CAD secondary prevention (De Backer, 2023; Le Grand et al., 2023).

These discoveries not only construct molecular frameworks for understanding CAD recanalization barriers but also establish theoretical foundations for developing ECM-targeted therapies and recurrence risk stratification strategies.

## 7 Animal models and translational research

**FIGURE 3:**
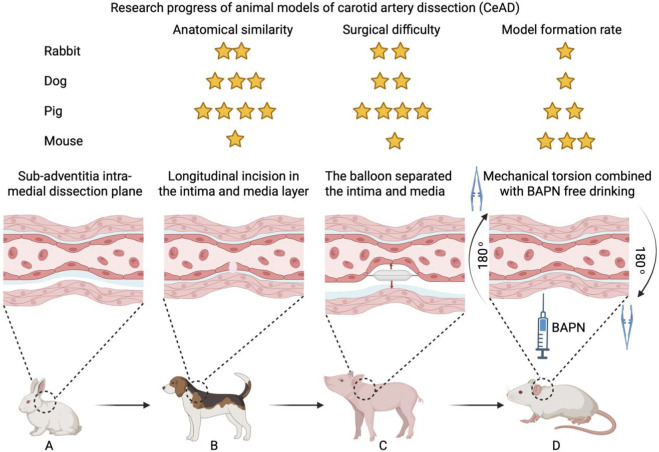
**(A–D)** Evolution of CAD animal models.

The absence of successful CAD models has perpetuated substantial uncertainty and controversy regarding optimal treatment strategies. Consequently, developing pathophysiologically relevant models represents an urgent research priority. Some researchers established a New Zealand white rabbit model featuring surgically created subadventitial dissection planes in internal carotid arteries (Kahler and Stuart, 1998), (See Figure 3A). However, absence of arterial thrombosis or stenosis during secondary observations limited human disease relevance. An experiment on a rabbit model of aneurysm induced by elastic elastin provided us with a reproducible model and similar hemodynamic characteristics to human cerebral arteries, which offered inspiration for our future exploration of the efficacy of new vascular endovascular devices (Wang and Yuan, 2012). Subsequently, another person generated elliptical intimal-medial defects or longitudinal incisions in canine common carotid arteries, producing aneurysmal dilatation or stenosis (Okamoto et al., 2002), (See Figure 3B). This study suggested intimal entry zone dimensions determine morphological changes post-experimental CAD. Given substantial interspecies differences, these models inadequately recapitulate human pathophysiology. Porcine common carotid diameters (4–5 mm) closely approximate human dimensions (4–6 mm), making porcine models potentially superior for human CAD simulation (Fujimoto et al., 2013). Thus, a CAD model was established using separators and balloon dilation in mini-pigs. This model can be easily adapted to individual research designs and investigate various possible intervention measures. It will become a useful tool for CAD pathophysiological translational research in the future (Peng et al., 2021), (See Figure 3C). Technical complexity, however, limits widespread adoption. Therefore, a CAD rat model based on mechanical torsion combined with BAPN (beta-aminopropionitrile) was proposed (Zhang et al., 2024), (See Figure 3D). This model may facilitate advanced CAD recanalization research. As vascular wall structural and functional integrity disruption constitutes essential pathological changes in arterial dissection, the lack of animal models accurately replicating human CAD pathophysiology remains a critical research gap—suggesting these models may serve as innovative platforms for future investigations.

## 8 Future directions for CAD recanalization

Future CAD recanalization research demands interdisciplinary integration and precision medicine paradigm innovation. Multimodal imaging technologies—incorporating high-resolution MRI, photon-counting CTA, intravascular ultrasound, and optical coherence tomography (OCT)—combined with dynamic 3D modeling and hemodynamic analysis enable quantitative assessment of dissection morphology, thrombus burden, and vascular remodeling potential. Artificial intelligence-driven radiomics may further enhance early high-risk lesion identification and dynamic recanalization strategy navigation, providing real-time decision support for personalized therapy.

Regarding pathological mechanisms, future investigations should explore CAD pathogenesis through vascular endothelial injury, inflammatory microenvironment imbalance, and extracellular matrix remodeling molecular networks—employing multi-omics approaches to identify potential recanalization mechanisms. Large-scale multicenter randomized controlled trials (RCTs) must clarify therapeutic efficacy differences across demographic subgroups (sex, age strata, dissection classifications) to establish stratified intervention windows and standardized outcome assessment frameworks, ultimately resolving current clinical controversies.

Endovascular technological innovation should leverage bioabsorbable stents, directional thrombolysis catheters, and other device advancements—combined with decade-long prospective cohort data—to systematically optimize restenosis and thromboembolic complication prevention strategies. Adjunctive stem cell transplantation or localized gene delivery may achieve functional vascular wall regeneration.

Furthermore, the integration of recanalization risk factors, imaging features, molecular biomarkers, and longitudinal follow-up data may facilitate the development of artificial intelligence–based predictive models—offer a robust foundation for evidence-based approaches to CAD recanalization while addressing current clinical challenges through effective solutions.

## 9 Conclusion

CAD recanalization fundamentally represents a dynamic equilibrium between vascular wall injury repair and pathological remodeling. Through integrated multimodal imaging quantification, molecular mechanism elucidation, and AI predictive modeling, current clinical management bottlenecks may be overcome—advancing precision medicine applications in vascular diseases. Both conservative and surgical CAD management can yield favorable outcomes. While conservative approaches remain first-line with demonstrated efficacy, surgical intervention proves beneficial and safe for select cases unresponsive to medical therapy. Future refined clinical classification systems coupled with large population studies may yield transformative breakthroughs in CAD recanalization.
